# Motivational and Behavioral Activation as an Adjunct to Psychiatric Rehabilitation for Mild to Moderate Negative Symptoms in Individuals with Schizophrenia: A Proof-of-Concept Pilot Study

**DOI:** 10.3389/fpsyg.2016.01759

**Published:** 2016-11-14

**Authors:** Kee-Hong Choi, Eunju Jaekal, Ga-Young Lee

**Affiliations:** Department of Psychology, Korea UniversitySeoul, Republic of Korea

**Keywords:** behavioral activation, motivational interviewing, psychosocial intervention, negative symptoms, schizophrenia

## Abstract

Few psychosocial approaches address the negative symptoms of schizophrenia, which shares common features with depression and anxiety. Behavioral activation (BA) is effective for addressing depression and anxiety in adults with various mental disorders. Motivational interviewing (MI) has been successfully applied to address ambivalence or lack of motivation toward treatment. Motivational and behavioral activation (mBA) has been developed by incorporating the core principles from BA and MI with recent findings on the negative symptoms of schizophrenia. In this study, we aimed to examine the feasibility and preliminary efficacy of mBA in a non-randomized controlled pilot study that included individuals with schizophrenia with mild to moderate negative symptoms receiving psychiatric rehabilitation. A total of 73 individuals with schizophrenia were recruited. Forty-seven of the participants who met the study inclusion and exclusion criteria were assigned to either an mBA + usual psychiatric rehabilitation group (mBA) or a usual psychiatric rehabilitation only group (treatment as usual, TAU). Administering mBA to individuals with schizophrenia with mild to moderate negative symptoms was feasible in a community mental health setting. Relative to TAU, mBA was associated with large effects in reducing negative symptoms measured using the Positive and Negative Syndrome Scale (PANSS) and the Brief Negative Symptom Scale (BNSS). However, after considering PANSS cognitive deficits and marital status as covariates due to significant differences in their baseline levels, the treatment effects on the BNSS were partially observed. In addition, participants in the mBA group showed improved verbal learning and memory compared with those in the TAU group. In individuals with schizophrenia receiving the usual forms of psychiatric rehabilitation in a community mental health setting, mBA appears to offer a promising adjunctive approach for addressing mild to moderate negative symptoms. Further investigations are needed to replicate the current findings in a randomized controlled trial.

## Introduction

Many individuals with schizophrenia experience negative symptoms, which are a key determinant of poor functioning and quality of life (Pogue-Geile and Harrow, 1985; Rabinowitz et al., 2012). Negative symptoms can persist even in individuals who participate in the usual forms of psychiatric rehabilitation offered in the community (Buchanan, 2007). Both clinicians and participants would benefit from a psychosocial treatment approach that targets persistent negative symptoms and can easily be delivered in community mental health settings. However, the majority of psychosocial interventions focus primarily on positive symptoms (Breier et al., 1991; Milev et al., 2005). Few studies have reported the efficacy of psychosocial treatments for negative symptoms (Dobson et al., 1995; Rector and Beck, 2001; Tarrier et al., 2004; Kurtz and Mueser, 2008; Staring et al., 2013; Velligan et al., 2015), and the estimated effect size reported in a recent meta-analysis was small and inconsistent across studies (Velthorst et al., 2015). Another meta-analysis indicates that social skills training had small to moderate effects (*d* = 0.40; 95% CI = 0.19, 0.61) on negative symptoms; although, the benefits only appear to be stable for younger patients with schizophrenia or in studies of greater design quality (Kurtz and Mueser, 2008). Most recently, Velligan et al. (2015) reported potential treatment gains using comprehensive psychosocial treatments for those who have persistent and clinically significant negative symptoms. Thus, the effective and cost-efficient treatment of negative symptoms is a high priority when developing interventions.

A two-factor model of negative symptoms describing experiential (avolition, asociality, and anhedonia) and emotional expressive impairments (alogia and flat affect) has been shown to better explain the heterogeneity of negative symptoms in schizophrenia than a single factor model (Horan et al., 2011; Kirkpatrick et al., 2011; Jang et al., 2016a). Due to the establishment of a vicious cycle between the two distinct but related domains of negative symptoms, individuals with schizophrenia not only exhibit low base rates of behavior and have reduced rates of positive reinforcement from the environment (Silverstein, 2010), but also lack the opportunity to identify, estimate the effort required, and pursue their own values and goals (Gold et al., 2008, 2013; Strauss et al., 2011).

Even though cognitive behavioral therapy for psychosis (CBT-p) and CBT for negative symptoms (CBT-n) take comprehensive approaches to addressing diverse problems including positive symptoms, depressive symptoms, dysfunctional beliefs (e.g., defeatist beliefs), and negative symptoms (Morrison and Barratt, 2010; Grant et al., 2012; Staring et al., 2013), we aimed to exclusively target a two-factor model of negative symptoms using the core principles of behavioral activation (BA) and motivational interviewing (MI).

BA is an evidence-based treatment option for depression (Cuijpers et al., 2007; Mazzucchelli et al., 2009), and might be a treatment option for negative symptoms. BA may interrupt the vicious cycle by assisting individuals with schizophrenia to reconnect with positive experiences using daily activity monitoring, values and goals assessment, and goal-led activity scheduling. More importantly, BA would be a cost-efficient option for both clinicians and individuals with schizophrenia mainly due to its concise structure and principles (Porter et al., 2004; Ekers et al., 2011). Porter et al. (2004) suggested that group-format BA is a viable and effective adjunct to the usual treatment conducted in community mental health settings for depressed clients. Ekers et al. (2011) demonstrated that generic mental health professionals without expertise in psychotherapy could deliver BA in an effective manner.

Recent neuroscience data indicate that individuals with schizophrenia have an intact capacity for experiencing here-and-now pleasure, but experience difficulty recalling and predicting pleasure (i.e., retrospective and prospective hedonia) with aberrant attentional deficits to pleasurable stimuli (Strauss and Gold, 2012; Jang et al., 2016b,c). Thus, it is speculated that daily activity monitoring would help to allocate attention to meaningful and pleasant daily activities that individuals with schizophrenia have had in the recent past. At the beginning of each session, there is an additional opportunity to recall and share the participant’s pleasurable activities in the past week without looking at their monitoring form. This was used to enhance the participant’s retrospective hedonic experience. Assessments of one’s own values and goals and implementation of value/goal-led activities would help to link here-and-now pleasure with the prediction of pleasure for prospective hedonistic experiences. Given the difficulties that individuals with schizophrenia have in goal-setting and planning, several goal domains (i.e., interpersonal, educational, vocational, independent living, etc.) were provided to them in printed form. Specific activities (i.e., less than 5) under each goal domain were also provided in a summarized form so that participants could voluntarily choose among the options. Therapists also encouraged the participants to link each activity with their own goals, and encouraged moving slowly to the other goals and activities that they had not yet explored. During this process, given the motivational deficits of individuals with schizophrenia, MI becomes a core principle and is the core method used (McCambridge and Strang, 2003).

Several adjustments were made to the original BA. These included simplification of treatment materials (e.g., monitoring and scheduling forms) and terms, providing greater structure for every session, minimizing the number of forms to be completed during the session, minimizing homework activities, developing strategies to remember core activities (i.e., monitoring and activating activities), slowing the pace of conversation, and greater repetition for those with cognitive impairments.

Recent meta-analysis indicates that social skills training is associated with some improvement in negative symptoms (Kurtz and Mueser, 2008). In each session, brief components of social skills training that could be delivered in the context of the motivational and behavioral activation (mBA) group session [i.e., speaking loud enough to be heard, practicing (half) smiles while talking about pleasant events, listening to others, and providing feedback to others] were incorporated to target emotional expressivity (e.g., vocal expression, expressive gestures, facial expression, quantity of speech). The clinicians then provide consistent positive feedback whenever the participants display emotional expressivity (Supplementary Table S2).

After the incorporation of these adjustments, BA could assist individuals with schizophrenia to recall and monitor pleasurable and goal-directed activities in the past and to plan and implement a future schedule. Given extensive data supporting the efficacy of behaviorally oriented approaches in psychiatric rehabilitation (e.g., contingency management) for individuals with schizophrenia with various levels of functioning (Porter et al., 2004; Ekers et al., 2011; Granholm et al., 2015; Murphy et al., 2015), BA would be feasible and beneficial for individuals with schizophrenia with mild to moderate negative symptoms. Mairs et al. (2011) reported in a small-sample pilot case study that BA exhibited a large effect and appeared to be a promising approach for treating the negative symptoms of schizophrenia.

To target motivational deficits or ambivalence toward treatment, MI has recently been successfully combined with various interventions such as cognitive remediation (Rüsch and Corrigan, 2002; Steinberg et al., 2004; Fiszdon et al., 2016), and has demonstrated synergistic effects. Likewise, MI is incorporated into BA to better understand the relationships between ambivalence about change and each participant’s goals, values, and resistance. It is carried out by expressing empathy, rolling with resistance, supporting self-efficacy, and developing discrepancy whenever necessary (Miller and Rollnick, 2002). MI is a particularly important component when assisting the participant in his/her search for their own goals/values, potential barriers and concerns, and for evaluating and establishing solutions and plans (McCambridge and Strang, 2003). Since BA and MI share common aspects (e.g., searching for values/goals and planning for behavioral changes), MI is not only well integrated into BA, but is expected to further help to deliver the core components of BA to individuals with schizophrenia with motivational deficits or ambivalence toward behavioral change.

With these theories and findings in mind, we have developed the mBA Program as an adjunct to psychiatric rehabilitation for individuals with schizophrenia with mild to moderate negative symptoms. This is a brief manual-based psychological approach that incorporates BA principles and MI components targeting a two-factor model of negative symptoms.

For Stage I-A of intervention development, as suggested by Onken et al. (2014), the authors translated and modified the Brief Behavioral Activation Treatment for Depression (BATD) manual by Lejuez et al. (2001) and evaluated its feasibility in eight community-dwelling individuals with schizophrenia with mild to moderate negative symptoms using a 12-session group format (Choi et al., 2014), with a low dropout rate (*n* = 1, employed). Following Stage I-A, the authors incorporated feedback from participants and clinicians, and the program was revised accordingly, with the BATD manual being simplified to a 10-session group format (Choi et al., 2014). With these revisions, the mBA program was finally established for a Stage I-B pilot trial.

We hypothesized that the mBA program would be feasible and tolerable to individuals with schizophrenia with mild or moderate negative symptoms participating in community-based psychiatric rehabilitation. In addition, it was hypothesized that the mBA group would have lower levels of negative symptoms compared with the treatment as usual (TAU) group. Lastly, given that extensive research supports the link between negative symptoms, daily functioning, and cognitive impairments (Green, 1996; Velligan et al., 1997; Green et al., 2000; Foussias and Remington, 2010), and the mBA program includes repetitive practice of attending and recalling one’s own activities and planning, we explored whether mBA was associated with recovery in neurocognition.

## Materials and Methods

### Participants

Seventy-three participants were recruited from community mental health centers and day hospitals from September 2012 until January 2015. Inclusion criteria for the participants were as follows (Buchanan, 2007): (1) a primary diagnosis of schizophrenia or schizoaffective disorder, (2) aged between 18 and 65 at the time of pre-testing, (3) mild to moderate negative symptoms [greater than 3 on at least two negative symptom items from the Positive and Negative Syndrome Scale (PANSS)], and (4) stably prescribed psychotropic medications for at least the previous 6 months. Exclusion criteria included (1) a history of organic brain syndrome, seizures, or traumatic brain injury, (2) current comorbid disorders such as mental retardation, or current alcohol or other substance abuse/dependence, (3) more than moderate positive symptoms (greater than 4 on PANSS positive symptom items), (4) more than mild depressive symptoms (greater than 3 on PANSS depressive symptom items), (5) receiving psychological services that include behavioral activation components, and (6) risk of self-harming or homicide. There were no differences in demographic variables between the two groups, except for marital status and baseline PANSS cognitive symptoms (**Table 1**), which were included as covariates in subsequent analyses (**Table 1**).

**Table 1:** Baseline characteristics of participants.

	Treatment	Control group	*t* or χ^2^
	group (*n* = 23)	(*n* = 24)	
Age, *M* (*SD*)	40.43 (11.72)	44.38 (10.77)	-1.20
Age of onset, *M* (*SD*)	24.22 (7.03)	24.29 (8.39)	-0.03
Years of education, *M* (*SD*)	12.04 (2.67)	11.21 (3.58)	0.90
Gender, *n* (%)			
Men	11 (47.83)	12 (50.00)	0.02
Women	12 (52.17)	12 (50.00)	
**Marital status, *n* (%)**			
Married	1 (4.35)	9 (37.50)	11.05^∗∗^
Single	18 (78.26)	8 (33.33)	
Divorced/other	4 (17.39)	7 (29.17)	
Medication, *n* (%)	23 (100.00)	24 (100.00)	–
**PANSS, *M* (SD)**			
Negative	18.17 (3.66)	18.38 (4.21)	-0.17
Excitement	6.52 (1.93)	6.83 (2.06)	-0.54
Cognitive	11.22 (2.56)	13.08 (3.36)	-2.14^∗^
Positive	8.57 (2.76)	8.46 (3.48)	0.12
Depression	9.52 (2.76)	9.17 (3.12)	0.41
**Total**	68.26 (9.86)	69.67 (14.13)	-0.39

*^∗^*p* < 0.05; ^∗∗^*p* < 0.01.*

### Procedures

**FIGURE 1:**
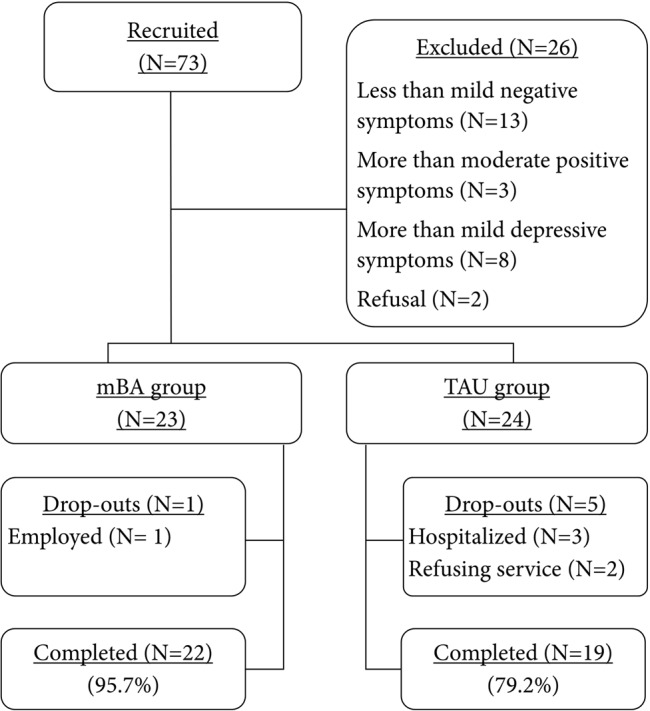
**Flow chart of participants**.

The participants in this pilot study were recruited from various community mental health centers located in urban areas in South Korea. Prior to pre-treatment assessment, all participants provided informed consent. Local Institutional Review Boards approved this study. After the pre-treatment assessment, 47 participants who met study inclusion criteria were allocated to either the mBA (*n* = 23) or the TAU group (*n* = 24) (**Figure 1**) based on negative symptom scores. Group allocation was made alternately from active group to control group, and thus the most subjects were assigned to the active group or the control group based on their interest to participate in the current study after having received information on the study. Regardless of the allocated group, participants attended the centers for psychiatric rehabilitation program. About 10 weeks after completing the mBA program, the post-treatment assessment was administered to mBA and TAU groups over similar time frames. Of the 49 participants, one participant (of 23) in the treatment group was dropped due to job conflicts, while five participants (of 24) in the control group were dropped for reasons such as hospitalization, service refusal, and losing contact.

### Motivational and Behavioral Activation

The mBA program for negative symptoms was provided by the researchers in our team. Fidelity was assessed with a random visit (92.31%; Supplementary Table S1). Participants received ten 60- to 70-minute group sessions either once or twice weekly. The main purpose of mBA was to increase the level of routine and social activities that participants identified as pleasurable, meaningful, and valuable. Specifically, mBA assisted participants (1) to monitor daily activities in the previous week, (2) to set goals, (3) to identify and plan daily activities that fit their own goals, (4) to administer their own goal-led activities, and (5) to identify and address barriers to achieving their goal-led activities. Throughout the mBA program, participants were expected to practice (1) goal setting, (2) monitoring of activities and remembering pleasurable and meaningful activities, (3) goal-led activity planning, and (4) problem-solving skills. Therapists were expected to persistently use MI techniques such as employing open-ended questions, using positive feedback, reflection, and summarizing the participant’s words, especially for participants with ambivalence or lack of motivation toward the treatment. In addition to target emotional expressivity in the context of mBA, brief components of social skills training [i.e., speaking loud enough to be heard, practicing (half) smiles while talking about pleasant events, listening to others, and providing feedback to others] were practiced in each session. The content of each session of mBA is presented in Supplementary Table S2.

### Measures

Participants each completed a semi-structured interview-based psychiatric symptom assessment, self-report questionnaires for motivation, and brief neurocognitive assessment batteries. All interviews were conducted by licensed clinical psychologists and clinical psychology graduate students who obtained satisfactory intra-class correlations (ICCs).

#### Psychiatric Symptoms

The PANSS (Kay et al., 1987) was administered to evaluate the severity of psychiatric symptoms. Each item was scored on a 1 (absent symptom) to 7 (extreme symptom) Likert scale. Five factor scores were calculated for the PANSS, i.e., Negative symptoms, Excitement, Cognition, Positive symptoms, and Depression (Bell et al., 1994; Lindenmayer et al., 1994, 1995). In the current study, Cronbach’s α for each subscale was as follows: Negative symptoms = 0.87, Excitement = 0.64, Cognition = 0.69, Positive symptoms = 0.70, and Depression = 0.67. ICCs for each subscale were as follows: Negative symptoms = 0.93, Excitement = 0.69, Cognition = 0.95, Positive symptoms = 0.98, Depression = 0.50.

The Brief Negative Symptom Scale (BNSS) is a semi-structured clinical interview that measures the severity of negative symptoms in schizophrenia and schizoaffective disorder (Kirkpatrick et al., 2011). The BNSS was based on a two-factor model of negative symptoms, consisting of experiential impairment (anhedonia, asociality, and avolition) and expressive impairment (alogia and blunted affect) (Horan et al., 2011; Strauss et al., 2012). Thirteen items, each ranging from absence of symptom (0) to extremely severe symptom (6) in motivation–pleasure and emotional expressivity subscales, were assessed over a time period of 1 week. Since the BNSS was translated into Korean and was available after the initiation of this trial, we obtained BNSS data from thirty participants (*n* = 14 for mBA, *n* = 16 for TAU). In the current study, Cronbach’s α for the motivation–pleasure subscale was 0.93 and for the emotional expressivity subscale was 0.86. ICCs for each subscale were as follows: motivation–pleasure = 0.68, emotional expressivity = 0.89.

#### Neurocognitive Function

The Korean Auditory Verbal Learning Test (K-AVLT) was designed to evaluate short-term memory, auditory verbal memory, and learning strategies (Rey, 1964). Cheong et al. (1999) revised and validated the Korean version. After listening to 15 words presented verbally, participants were asked to complete free recall trials immediately and after a 30-minute delay, and a recognition trial.

Trail Making Test A and B (TMT-A, B) are included in the Halstead–Reitan neuropsychological test battery (Reitan, 1992). In part A, subjects were asked to sequentially connect dispersed digits (1–25) to measure psychomotor speed and attention. In part B, subjects were required to connect 15 digits and 14 letters alternately in ascending order, to measure executive function related to frontal lobe activity such as mental flexibility and visuospatial working memory. Times to complete the tasks and the number of errors constituted the test scores. If subjects exceeded 90 s in part A or 300 s in B, the data were excluded from the analysis.

The Coding (CD) subtest in the Wechsler Adult Intelligence Scale–Fourth Edition (WAIS-IV; Wechsler, 2008; Hwang et al., 2012) was used to measure visual-motor coordination, persistence of concentration, and short-term visual memory. Subjects were required to copy as many symbols as possible within 120 s using a key. Total scores were based on the number of correctly matched items.

#### Premorbid Intelligence Quotients

To estimate premorbid IQ, the Information subtest (IN) in the K-WAIS-IV (Hwang et al., 2012) was administered. Items covered a range of common, realistic, and general knowledge. Premorbid intelligence was estimated using an algorithm from the Korea Premorbid Intelligence Estimate for the WAIS-IV (KPIE-IV; Kim et al., 2015).

## Results

### Feasibility

Dropout rates (4.35% for mBA vs. 20.83% for TAU) were similar between the mBA and the TAU groups (χ^2^ = 2.87, *p* = 0.09), indicating that mBA would be tolerable and feasible to administer to individuals with schizophrenia with mild to moderate negative symptoms participating in the usual psychiatric rehabilitation services.

### Effects of mBA on Psychiatric Symptoms

We conducted repeated-measures ANOVAs for negative symptom scores and other psychiatric symptoms (i.e., positive, depressive, cognitive, and excitement symptoms) measured using the PANSS and the BNSS, with treatment and control groups as the between-subjects factor (mBA vs. TAU) and time as the within-subjects factor (pre- and post-) (**Table 2**). These analyses revealed no significant main effect of group on any PANSS subscale except for the negative scale, *F*_(1,39)_ = 4.15, *p* < 0.05, and cognitive scale, *F*_(1,39)_ = 23.87, *p* < 0.001. There were significant interaction effects of group by time on scores for the negative symptoms subscale, *F*_(1,39)_ = 10.10, *p* < 0.01, cognitive symptoms subscale, *F*_(1,39)_ = 10.45, *p* < 0.01, and depression subscale, *F*_(1,39)_ = 10.99, *p* < 0.01 (**Table 2**). The findings indicate that participants in the mBA improved their negative, cognitive, and depressive symptoms compared to those in the TAU group. Even after considering baseline PANSS cognitive symptoms or marital status as a covariate, all of the significant findings reported above were maintained.

**Table 2:** Effects of mBA on psychiatric symptoms.

Measure	Group	Pre	Post	Main effect		Interaction effect	Effect Size
		*M* (*SD*)	*M* (*SD*)	Group *F*	Time *F*	Group × time *F*	Partial η^2^
**PANSS^a^**						
Negative	Treatment	18.05 (3.70)	14.09 (4.74)	4.15^∗^	12.49^∗∗^	10.10^∗∗^	0.21
	Control	18.79 (4.50)	18.58 (5.07)				
Excitement	Treatment	6.50 (1.97)	6.55 (2.13)	0.48	0.48	0.33	0.01
	Control	6.63 (1.80)	7.11 (2.05)				
Cognitive	Treatment	11.36 (2.52)	9.82 (2.68)	23.87^∗∗∗^	0.21	10.45^∗∗^	0.21
	Control	13.00 (3.04)	15.05 (3.24)				
Positive	Treatment	8.68 (2.77)	9.00 (3.34)	0.01	3.15	1.50	0.04
	Control	7.90 (2.94)	9.63 (3.42)				
Depression	Treatment	9.59 (2.81)	8.96 (2.79)	0.75	3.56	10.99^∗∗^	0.22
	Control	8.90 (3.36)	11.21 (3.87)				
**BNSS^c^**						
Motivation and pleasure	Treatment	21.29 (8.67)	16.86 (7.86)	1.07	3.56	4.72^∗a^	0.14
	Control	21.69 (7.60)	22.00 (7.53)				
Emotional expressivity	Treatment	18.50 (5.97)	15.29 (7.17)	0.12	0.84	4.35^∗b^	0.14
	Control	17.13 (8.35)	18.38 (7.36)				

*^∗^*p* < 0.05; ^∗∗^*p* < 0.01; ^∗∗∗^*p* < 0.001.*

*^a^After considering baseline PANSS cognitive symptoms as a covariate, the treatment effect on the BNSS motivation and pleasure subscale disappeared (*p* = 0.12).*

*^b^The treatment effect on the BNSS subscales disappeared (*n* = 11 for mBA, *n* = 5 for TAU) when examining the study hypotheses only using participants with single marital status.*

*^c^BNSS sample size (*n* = 14 for mBA, *n* = 16 for TAU).*

In addition, there was no significant main effect of group on the BNSS score. However, there were significant interaction effects of group by time on the motivation and pleasure subscale, *F*_(1,28)_ = 4.72, *p* < 0.05, and emotional expressivity subscale, *F*_(1,28)_ = 4.35, *p* < 0.05 (**Table 2**). The findings indicate that participants in the mBA group improved their BNSS subscale scores when compared to those in the TAU group. However, after considering baseline PANSS cognitive symptoms as a covariate, the treatment effect on BNSS motivation and pleasure subscales disappeared (*p* = 0.12). To consider marital status as a covariate, we examined the study hypotheses only using participants who were single. Using these participants, the treatment effects on the BNSS subscales disappeared (*n* = 11 for mBA; *n* = 5 for TAU).

To delineate the significant interaction effects, we calculated a partial eta-squared measure of effect size for use in ANOVA (Cohen, 1988; Miles and Shevlin, 2001) with general rules of thumb for the magnitude of the effect sizes, i.e., small (∼0.01), medium (∼0.06), and large (∼0.14). The effect sizes for the treatment group were mostly large for the above-mentioned negative symptoms, cognition, and depressive symptoms in the PANSS and BNSS (**Table 2**).

### Effects of mBA on Neurocognitive Function

We conducted repeated-measures ANOVAs on neurocognitive function with the treatment and control groups as the between-subjects factor (Group) and time (pre-, post-) as the within-subjects factor (**Table 3**). The analyses revealed no significant main effect of group on neurocognitive function. There was a significant interaction effect of group by time on the K-AVLT total score, *F*_(1,39)_ = 5.37, *p* < 0.01 (**Table 2**). The effect size for the treatment group on the K-AVLT total score was medium (**Table 3**).

**Table 3:** Effects of mBA on neurocognitive function.

Measure	Group	Pre	Post	Main effect		Interaction effect	Effect size
		*M* (*SD*)	*M* (*SD*)	Group *F*	Time *F*	Group × time *F*	Partial η^2^
Coding	Treatment	34.37 (7.73)	37.42 (8.85)	0.18	12.18^∗∗^	0.00	0.00
	Control	35.45 (8.70)	38.53 (8.06)				
Digit span	Treatment	44.05 (10.18)	44.42 (9.60)	1.11	4.05	2.50	0.07
	Control	39.49 (10.37)	42.56 (8.75)				
K-AVLT total	Treatment	28.95 (13.19)	41.21 (16.44)	2.16	14.30^∗∗^	5.37^∗^	0.13
	Control	28.11 (10.79)	31.05 (11.24)				
TMT-A	Treatment	38.18 (11.76)	43.28 (10.26)	0.39	7.82^∗∗^	0.02	0.00
	Control	35.74 (16.87)	40.34 (16.83)				
TMT-B	Treatment	39.46 (15.65)	41.82 (16.53)	2.10	0.03	0.43	0.01
	Control	32.21 (22.39)	30.79 (24.27)				

*^∗^*p* < 0.05; ^∗∗^*p* < 0.01.*

## Discussion

We have developed a manual-based brief psychosocial intervention that incorporates the core principles of BA and MI with recent findings on negative symptoms to target the two-factor components of negative symptoms in schizophrenia. The current study is the first to investigate the feasibility and efficacy of mBA as an adjunct to psychiatric rehabilitation for individuals with schizophrenia with mild to moderate negative symptoms, compared with TAU. In accordance with Onken et al. (2014), the current study is considered to be Stage I-B of an intervention development process.

The results indicate that the dropout rate for mBA was minimal compared with TAU (4.35% for mBA; 20.83% for TAU). This may support its feasibility for community dwelling individuals with schizophrenia. Velligan et al. (2015) reported a dropout rate of 34.6% in their recent randomized controlled trial (RCT) of the MOtiVation and Enhancement (MOVE) program conducted over 9 months. Given that the MOVE trial targeted persistent and clinically significant negative symptoms in a comprehensive manner over a longer treatment period, it is speculated that the concise structure of mBA (e.g., principles and techniques), the shorter treatment duration, and the participant characteristics (mild to moderate negative symptoms, regular participation in psychiatric rehabilitation program) would permit greater treatment survival rates. Even though a 10-session time frame is quite challenging for tackling negative symptoms, the repetition of exercises and gradual addition of treatment components across sessions appeared to provide enough opportunities for gaining treatment effects. Another possible reason for the differential drop-out rates between the mBA and TAU groups may be the potential extraneous variable affecting the allocation of the participants, as this was a non-randomized trial and the mBA and TAU groups were formed alternately. Thus, in this procedure, participants might have known the group they were allocated to prior to group allocation. This may have resulted in selection bias; for example, participants who had greater motivation for change may have been more likely to be assigned to the mBA group than the TAU group. To examine the potential impacts of different demographic characteristics (i.e., marital status) and psychiatric conditions (i.e., PANSS cognitive impairments factor) on drop-out rates, we compared those who dropped out to those who did not drop-out in terms of demographic and symptom variables. There were no differences in age, gender, age of onset, years of education, and PANSS subscales. The only observed difference was in marital status (χ^2^ = 10.755, *p* = 0.005). Interestingly, none of the single participants dropped out of either group. Even though marital status was not related to the primary study findings, a future study should examine the relationship between marital status and drop-out rates in the mBA.

As hypothesized, mBA was associated with large treatment effects on negative symptoms, cognition, and depressive symptoms in the PANSS and the BNSS emotional expressivity subscale even after considering the PANSS cognitive factor. It is notable that the large effects gained within a shorter period (10 weeks) were comparable with those of other approaches conducted over a longer period (Kurtz and Mueser, 2008; Velligan et al., 2015; Velthorst et al., 2015). It is speculated that the comparable treatment gains might be attributable to the sample characteristic (mild to moderate negative symptoms) and/or treatment characteristics explicitly targeting a two-factor theory of negative symptoms.

After considering the PANSS cognitive factor, the treatment effects on the BNSS emotional expressivity were maintained but motivation and pleasure subscales disappeared. Since the BNSS was administered to a subsample of the study participants (*n* = 14 for mBA and *n* = 16 for TAU), this finding should be cautiously interpreted because of its lack of power in detecting potential therapeutic benefits. However, there are several possible interpretations. First, it is speculated that the PANSS cognitive factor might share common aspects (e.g., cognitive deficits) with motivation and pleasure (Bell et al., 1994; Foussias et al., 2014), rather than with emotional expressivity. Thus, in future studies, cognitive symptoms of allocated participants should be counter-balanced. Second, to target emotional expressivity, mBA incorporated brief components of social skills training [e.g., speaking loud enough to be heard, practicing (half) smiles while talking about pleasant events, listening to others, providing feedback to others, clinician’s positive and corrective feedback], of which repetitive practice in the context of BA group session may result in robust improvement on the BNSS emotional expressivity, even after considering cognitive symptoms. To clarify whether mBA would lead to incremental benefits beyond social skills training on the two distinct factors of negative symptoms, mBA should be compared with social skills training in a future study.

In addition, even though we recruited participants with mild to moderate negative symptoms and less-than-mild depressive symptoms to avoid comorbidity between negative symptoms and depressive symptoms, mBA was associated with improvement in depressive symptoms. Given that this is a preliminary study with limitations regarding the investigation of underlying mechanisms, common aspects between negative symptoms and depressive symptoms affected by mBA should be explored in a future study.

However, the current findings were gained from a Stage I-B pilot trial, and thus should be replicated in an RCT trial.

Interestingly, even though it was not specifically predicted, mBA was associated with a medium effect on verbal learning and memory measured by the K-AVLT total score. Even though this finding should be interpreted cautiously due to the absence of *a priori* hypothesis testing, it can be speculated that verbal learning and memory might mediate the treatment gains from mBA as they have functional implications in individuals with schizophrenia (Green et al., 2000) and depression (Clark et al., 2009). A future study should determine whether treatment components of the mBA (e.g., recalling pleasurable and meaningful activities) are related to improvements in verbal learning and memory.

Several limitations should be noted. With a non-RCT, the causality of mBA on outcome variables should not be assumed and needs to be replicated in a future RCT study. Second, the PANSS negative symptoms factor was used as an inclusion criterion for mild to moderate negative symptoms in the current study. However, the PANSS is often regarded as an inadequate measure of negative symptoms, especially for experiential negative symptoms. Although in our data, the PANSS negative symptom factor scores at baseline were strongly correlated with the BNSS motivation and pleasure subscale scores (*r* = 0.727, *p* < 0.001), in future trials of mBA, study inclusion criteria should be revised to assess for the primary target of mBA. Third, the current study did not include follow-up assessments. Thus, a future study needs to evaluate whether the treatment gains reported in the current study are maintained. Fourth, it should be determined whether the 10-session format is sufficient for mastery of the core treatment components by individuals with schizophrenia with mild to moderate negative symptoms. Even though the major purpose of the current study was to prove the concept and evaluate the preliminary efficacy, future trials should evaluate whether longer treatment sessions produce larger treatment gains. Finally, clinicians have reported that MI is a critical component while they are assisting and encouraging participants to search for their own goals/values, to link each activity with their own goals, and to encourage moving slowly to other unexplored goals and activities. However, there were no specific measures regarding these factors included in the study design. Thus, in a future study, specific scales (e.g., the Stage of Change Readiness and Treatment Engagement Scale) should be included to examine these phenomena.

Despite the limitations mentioned above, the current findings support the notion that mBA would be a promising adjunctive approach to the usual psychiatric rehabilitation services delivered in community mental health settings for addressing mild to moderate negative symptoms in individuals with schizophrenia. As a next step (Stage II), RCT trials should replicate the current findings to confirm the causality of mBA in improving mild to moderate negative symptoms. We believe that the current findings will facilitate the development of an effective psychosocial treatment for individuals with schizophrenia with mild to moderate negative symptoms who participate in community based psychiatric rehabilitation services.

## Author Contributions

K-HC designed the study and supervised overall research processes including assessment, data management, supervision of the research assistants, and wrote the first draft of the manuscript. EJ performed statistical analyses. EJ and G-YL conducted psychological assessment, diagnostic interviewing and psychiatric symptom ratings. All authors revised subsequent drafts of the manuscript. All authors contributed to and have approved the final manuscript.

## Conflict of Interest Statement

The authors declare that the research was conducted in the absence of any commercial or financial relationships that could be construed as a potential conflict of interest.

The reviewer DF and handling Editor declared their shared affiliation, and the handling Editor states that the process nevertheless met the standards of a fair and objective review.
