# Supplementary material for: Motivational and Behavioral Activation as an Adjunct to Psychiatric Rehabilitation for Mild to Moderate Negative Symptoms in Individuals with Schizophrenia: A Proof-of-Concept Pilot Study
Source: Front Psychol. 2016 Nov 14;7:1759. doi: 10.3389/fpsyg.2016.01759 (PMC5107574; doi:10.3389/fpsyg.2016.01759)
Supplement: Supplementary file 1 [file Table_1.DOCX]

Supplement Table 1. Fidelity Rating Checklist

| # | Questions | | Yes/No |
| --- | --- | --- | --- |
| 1 | Clinician competence | Clinicians have received training to conduct mBA |  |
| 2 |  | Clinicians have received training to conduct MI |  |
| 3 |  | Clinicians have access to manuals |  |
| 4 |  | Clinicians can operate independently during the sessions |  |
| 5 |  | Clinicians follow treatment manual during every session |  |
| 6 | Treatment materials | Participants are provided with treatment materials |  |
| 7 |  | Participants have easy access to treatment materials |  |
| 8 | Session  Fidelity | Clinicians provide information regarding the purpose of the treatment and link it to the participant’s goals and values |  |
| 9 |  | Participants practice emotional expressivity (e.g., speaking loud enough to be listened and appropriate facial expressions) |  |
| 10 |  | Participants monitor activities for the past week |  |
| 11 |  | Participants recall pleasurable and meaningful activities for the past week |  |
| 12 |  | Participants check the numbers of activities and draw them on a graph |  |
| 13 |  | Participants are provided with an activity checklist for each goal domain |  |
| 14 |  | Clinicians use motivational interviewing techniques (e.g., reflection, affirmation, open questions, summary, and showing empathy) |  |

* Note: mBA: motivational and behavioral activation; MI: motivational interviewing
