# Supplementary material for: Motivational and Behavioral Activation as an Adjunct to Psychiatric Rehabilitation for Mild to Moderate Negative Symptoms in Individuals with Schizophrenia: A Proof-of-Concept Pilot Study
Source: Front Psychol. 2016 Nov 14;7:1759. doi: 10.3389/fpsyg.2016.01759 (PMC5107574; doi:10.3389/fpsyg.2016.01759)
Supplement: Supplementary file 2 [file Table_2.DOCX]

Supplement Table 2. mBA session summary

| Session | Contents | Form | Activities |
| --- | --- | --- | --- |
| 1 | Psycho-education | PPT Slides | - Linking treatment goals with individual’s goals/values |
|  | Address ambivalence to change (if needed) | N/A | - Clinicians use MI techniques |
|  | Practice emotional expressivity | Rules summary | - Speaking loud enough to be listened - Practice (half) smiles while talking about pleasant events - Listen to others - Provide feedback to others |
|  | Monitor/recall past pleasurable experiences | Daily activity monitoring form (simplified from BATD manual) | - Monitor past pleasurable experiences based on personal memory |
| 2-3 | + Draw a graph to monitor/recall pleasurable activities | Daily activity monitoring form | - Monitor past pleasurable experiences based on personal memory - Draw a graph to highlight any changes |
|  | + Identify personal values/goals and link them to activities | Activity checklist under each goal | - Choose any activities from the activity checklist that they had forgotten about |
| 4 | + Plan pleasurable activities | Activity planning form | - Identify target activities - Specify where, when, with whom, and how - Identify potential problems/concerns |
| 5 | + Monitor positive moods | Daily activity monitoring form with monitoring moods section  Emotion words list | - Monitor past pleasurable experiences based on personal memory - Focus on moods while performing a specific activity |
| 6-9 | + Identify/activate under-activated area(s) | Daily activity monitoring form with monitoring moods section  Activity checklist under each goal | - Review activities and goals/values areas - Identify under-represented activities under each goal area and/or under-activated goal areas - Explore the participant’s interests in under-activated areas - Identify target activities - Specify where, when, with whom, and how - Identify potential problems/concerns |
| 10 | + Relapse prevention plan | Relapse prevention plan form | - Identify pleasant activities and goals/values that the participant has found - Ways to continue those activities - Potential barriers and concerns |
|  | + Provide certificate | Certificate for completion |  |

* Note: mBA: motivational and behavioral activation; MI: motivational interviewing; BATD: the brief behavioral activation treatment for depression (Lejuez et al., 2001)
